# Endocrine and Targeted Therapy for Hormone-Receptor-Positive, HER2-Negative Advanced Breast Cancer: Insights to Sequencing Treatment and Overcoming Resistance Based on Clinical Trials

**DOI:** 10.3389/fonc.2019.00510

**Published:** 2019-06-21

**Authors:** Rola El Sayed, Lara El Jamal, Sarah El Iskandarani, Jeries Kort, Mahmoud Abdel Salam, Hazem Assi

**Affiliations:** ^1^Oncology Division, Department of Internal Medicine, American University of Beirut Medical Center, American University of Beirut, Beirut, Lebanon; ^2^School of Medicine, American University of Beirut, Beirut, Lebanon

**Keywords:** advanced breast cancer, endocrine therapy, hormone receptor positive, HER2 negative, endocrine resistance, overcoming resistance, sequencing treatment

## Abstract

**Background:** Advanced hormone-receptor positive HER2 negative breast cancer is a common and a very heterogeneous disease. Hormone therapy is the main first line treatment of choice, given alone or in combination with other agents that have shown to improve patient outcomes, Nevertheless, treatment remains generally palliative rather than curative. Sequencing of such treatment remains challenging, especially with resurgence of variable resistance patterns. Multiple attempts have been made to overcome resistance and improve patient survival, yet resistance remains not very well understood and metastatic cancer remains a disease with dismal prognosis.

**Methods:** In this paper, we searched pubmed database as well as local and international meetings for all studies discussing advanced and metastatic hormone-receptor-positive, her2-negative breast cancer, hormonal treatment, resistance to hormonal treatment, mechanism of resistance, and means to overcome such resistance.

**Conclusion:** There does not exist an optimal treatment sequence for hormone-receptor-positive, her2-negative advanced breast cancer. However, after review of literature, a reasonable approach may be starting with tamoxifen, aromatase inhibitors, or fulvestrant in absence of visceral crisis, in addition to ensuring adequate ovarian function suppression in pre/peri-menopausal women. Aromatase inhibitors and fulvestrant seem to be superior. Resistance to such agents is increasing, mostly attributed to genetic and molecular changes. Multiple modalities are addressed to overcome such resistance including use of CKD4/6 inhibitors, mTOR inhibitors and PI3K inhibitors in addition to other agents under study, all with promising results. CDK4/6 inhibitors work best when used in frontline setting. Finally, treatment of breast cancer remains a growing field, and more studies are to be awaited.

## Introduction

Breast cancer is the most common malignancy and the second leading cause of cancer mortality among women worldwide (1). It is a heterogeneous disease with a variety of subtypes, each characterized by distinct clinical, pathologic and molecular features. It is becoming gold standard practice to classify a patient's breast cancer on the basis of its molecular features in order to determine prognosis and recommend treatment choices (2).

**Figure 1:**
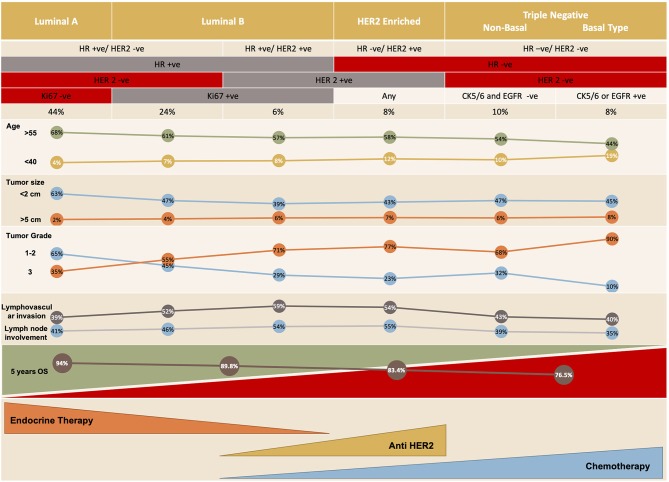
Breast cancer molecular subtypes (4, 5).

There are five distinct molecular subtypes of breast cancer: luminal A and B, human epidermal growth factor receptor 2 (HER2)-enriched, basal-like and claudin-low (3), with the last 2 being subcategories of triple-negative breast cancer (Figure 1). Each subtype can be targeted differently with systemic therapy. The available systemic therapies include endocrine therapy, targeted biologic treatment, chemotherapy, and best supportive care (6, 7); the recently investigated immunotherapeutic agent atezolizumab is also an option when combined with chemotherapy in the setting of triple-negative breast cancer. The choice of therapy is based on various criteria, including the patient's menopausal status, tumor markers, prior treatment (and response to that treatment), time to treatment failure, and the presence of metastases as well as patient co-morbidities (8).

The 5 years relative survival of individuals with breast cancer of all stages is 89.7% (9). However, the prognosis of patients with advanced metastatic breast cancer (MBC) is poor: their 5 years survival is approximately 26.9% (9). Advanced breast cancer (ABC) includes both metastatic and locally advanced disease. Although MBC remains an incurable disease, patients with hormone receptor (HR)-positive and HER2-negative ABC have better survival rates than other sub-categories of ABC (10, 11). About 75% of patients with MBC are HR positive, and more than 70% of these patients have HER2-negative tumors (12). Many clinical trials have focused on the treatment of this sub-category of breast cancer, in which endocrine therapy targeting HRs not only is an essential treatment option, but is highly recommended as the first-line treatment in the absence of immediately life-threatening situations or visceral crises. Endocrine therapy in the treatment of HR-positive ABC improves quality of life and has high efficacy with minimal toxicity (13).

The role of endocrine therapy in breast cancer has been established since early nineteenth century, with Mr. William Nunn noting improvement in breast lesions and regression of disease in peri-menopausal women with breast cancer after menopause, as well as Dr. George Thomas Beatson in the 1890s who stressed on the importance of hormone function suppression in breast cancer with his infamous oophorectomy series. Several decades later, in the 1970s, tamoxifen was brought to market and was found to increase survival. Since then, the landscape of the treatment of HR-positive breast cancer has been changing. There have been significant improvements in survival with anti-estrogen therapy, and a variety of drugs targeting multiple pathways have been developed, all with the promise of enhanced outcomes.

Clinical research is being increasingly directed toward trying to find biomarkers that predict a patient's response to different available therapeutic options, in conjunction with ongoing efforts to determine the optimal sequence of treatment approaches.

This review will compare the available endocrine therapy and targeted therapy options for HR-positive/HER2-negative ABC in postmenopausal women, with some mention of treatment of premenopausal women, considering the scarcity of data regarding pre-menopausal women, a population that is often under-represented in clinical trials. Therapy options include selective estrogen receptor (ER) modulators (SERMs), selective estrogen receptor degraders, aromatase inhibitors (AIs), mammalian target of rapamycin (mTOR) inhibitors, and cyclin-dependent kinase 4 and 6 (CDK 4/6) inhibitors, as well as other new options that are currently being explored. The mechanism of action of each therapy, its efficacy and safety, considerations for monotherapy vs. combination therapy, resistance modalities and means to overcome such resistance with newly available drugs will be discussed, as well as a suggestion of a possible optimal sequence of treatment.

## The Estrogen Receptor

**Figure 2:**
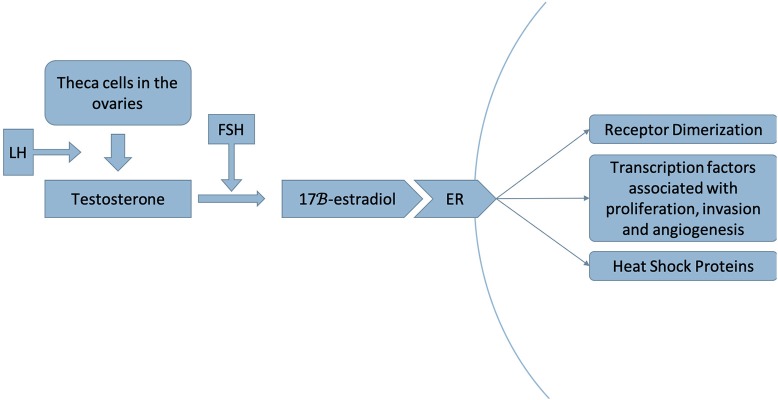
Luteinizing hormone (LH) stimulates the production of testosterone by theca cells in the ovaries. Testosterone is then converted to 17β-estradiol by the aromatase enzyme, a step that is stimulated by follicle-stimulating hormone (FSH) (17). This process occurs in adipose tissue as well as in muscle, liver, brain tissue and breast tumors (18). Estradiol acts as a ligand to the estrogen receptor (ER), dissociating the heat shock proteins from the receptor and inducing receptor dimerization, thereby activating a signaling pathway that recruits transcription factors associated with proliferation, invasion and angiogenesis of breast cancer (19).

**Figure 3:**
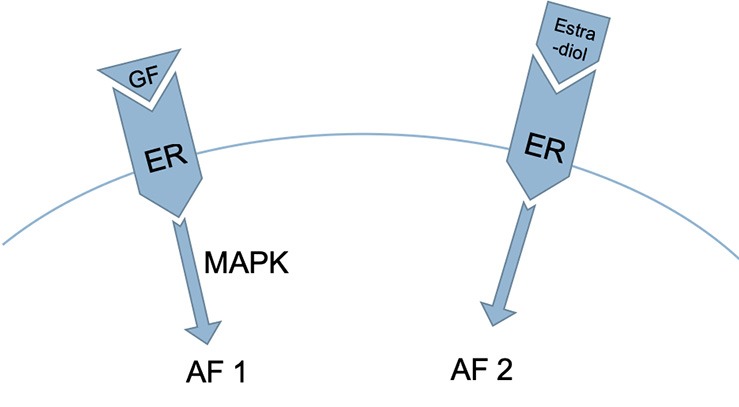
Two regions in the ER are involved in the process described in Figure 2: activation function 1 (AF1), which is activated by growth factors acting via the mitogen-activated protein kinase [MAPK] pathway, and activation function 2 (AF2), which is activated by estrogen (20, 21).

Luminal A and B breast cancers are ER-positive subtypes of breast cancer in which estrogen regulates and mediates cell growth. Increased exposure to estrogen from an intrinsic or extrinsic source is associated with an increased risk of breast cancer (14–16) (Figures 2, 3).

Two ERs have been discovered to date: ERα and ERβ (22). ERα is the major regulator of ER-positive breast cancer; ERβ's function is not well understood (23, 24).

Various expressed genes regulate the genomic action of the ER, including estrogen receptor 1 (ESR1), GATA-binding protein 3 (GATA3), forkhead box protein A1 (FOXA1) and runt-related transcription factor 1 (RUNX1). As a result, there is a high degree of heterogeneity among ER+ tumors.

The DNA binding sites of GATA3 and FOXA1 are located within the ESR1-binding regions. Lack of such binding sites hinders ER binding capacity and transcription activity (25). AI therapy produces a greater reduction in the Ki-67 proliferation marker in patients who have a GATA3 mutation than in those who do not (26), which demonstrates that a GATA3 mutation indicates a breast cancer that is sensitive to endocrine therapy. GATA3 and FOXA1 are mutated in a mutually exclusive manner, which suggests that a mutation in FOXA1 would also mark a breast cancer that is endocrine therapy sensitive. RUNX1 transcription factors on the other hand are tumor-suppressors in breast cancer, and their expression is decreased in aggressive types of breast cancer (27, 28). A mutation in RUNX1 is associated with resistance to AI therapy (26).

The ER signaling pathway, the phosphatidylinositol 3-kinase (PI3K)/protein kinase B (AKT)/mammalian target of rapamycin (mTOR) signaling pathway, the mitogen-activated protein kinase (CDK) 4/6-retinoblastoma (RB) pathway, the tumor protein p53 (TP53)/mouse double minute 2 homolog (MDM2) pathway and growth factor-receptor signaling pathways may be altered in luminal cancers that express the ER. Each one of these pathways may carry biomarkers that confer sensitivity or resistance to endocrine therapy. Moreover, modulation of these pathways can infer a means to bypass endocrine resistance. Effectors on these pathways will be discussed later in this article in the context of drugs that can be used in endocrine-resistant ABC before shifting to chemotherapy.

Table 1 summarizes some of the major randomized controlled trials of endocrine therapy in breast cancer.

**Table 1:** Endocrine therapy in ABC.

**Trial/ Phase**	**Intervention Comparison**	**T#**	**# pt**	**Pt characteristics**			**Disease characteristics**		**Previous therapy**		**Survival**			
				**Age**	**Menopausal status**									
					**Pre meno**	**Post meno**	**HR +ve**	**HER2 –ve**	**Chemo**	**Endocrine**	**OS**	**PFS or TTP**	**CBR**	**ORR**
**ENDOCRINE THERAPY**														
Di Leo et al. (29)	Fulvestrant 500 mg	2	374	61	0	100	100	NR	0	100	22.03	PFS, 5.5	39.6	NR
Phase 3	Fulvestrant 250 mg		362	61	0	100	100	NR	0	100	26.4	6.5	45.6	NR
	*p*										<0.05	<0.05	NR	
Howell et al. (30)	Fulvestrant 250 mg	1	313	67	0	100	78.9	NR	22.7	22	36.9	TTP, 6.8	54.3	31.6
Phase 3	Tamoxifen		274	66	0	100	77.4	NR	24.1	24.8	38.7	8.3	62	33.9
	*p*										0.04	0.088	0.026	0.45
Mouridsen et al. (31)	Letrozole	1	458	65	0	100	65	NR	30	19	34	TTP, 9.4	50	32
Phase 3	Tamoxifen		458	64	0	100	67	NR	34	18	30	6	38	21
	*p*										0.53	<0.0001	0.0004	0.0002
Robertson et al. (32)	Fulvestrant 500 mg	1	230	64	0	100	100	100	16	1	NR	PFS, 16.6	78	46
Phase 3	Anastrozole		232	62	0	100	100	100	19	<1	NR	13.8	74	45
	*p*											0.0486	0.3045	0.7290
Chia et al. (33)	Fulvestrant 500–>250	2	351	63	0	100	98.3	NR	24.8	89.2	NR	TTP, 3.7	32.2	NR
Phase 3	Exemestane		342	63	0	100	98.2	NR	21.6	86	NR	3.7	31.5	NR
	*p*											NS	NS	
Bergh et al. (34)	Anastrozole alone	1	256	63	NR	NR	97.7	NR	49.6	65.6	38.2	TTP, 10.2	NR	NR
Phase 3	Fulvestrant + anastrozole		258	65	NR	NR	98.4	NR	41.9	69.8	37.8	10.8	NR	NR
	*p*										NS	NS		
Mehta et al. (35) Phase 3	Anastrozole alone –> Fluvestrant	1	345	65	0	100	100	91.5	29.9	40	41.3	PFS, 13.5	70	NR
	Anastrozole + fulvestrant		349	65	0	100	100	89.6	37	40	47.7	15	73	NR
	*p*										0.05	0.05	NS	
Johnston et al. (36)	Fulvestrant + placebo	2	231	63.4	0	100	99.1	61/94	NR	100	19.4	PFS, 4.8	32	8
Phase 3	Fulvestrant + anastrozole		243	63.8	0	100	100	50/93	NR	100	20.2	4.4	34	8
	Exemestane		249	66	0	100	99.6	57/93	NR	100	21.6	3.4	27	4
	*P*										NS	NS	NS	NS

*T#, Treatment line; # pt, Number of patients; OS, Overall survival; PFS, Progression free survival; TTP, Time to progression; CBR, Clinical benefit rate; ORR, Objective response rate; NR, Not reported; NS, Not significant*.

## Selective Estrogen Receptor Modulators: Tamoxifen/Toremifene

Tamoxifen and toremifene are SERMs approved by the US Federal Drug Administration that block the activity of ERα (37). Once a SERM binds to the ER, it induces a conformational change in the receptor that leads to dimerization of the receptor and blockage of expression of estrogen-dependent genes. SERMs also reduce the activity of DNA polymerase and other enzymes (38), and induce the secretion of transforming growth factor β (TGF-β) within the mesenchyme of breast cancers (39), an autocrine and paracrine mediator that blocks cell proliferation (40). Moreover, tamoxifen has been demonstrated to decrease insulin-like growth factor I (IGF-I) levels in the serum, thereby decreasing mitosis in breast cancer cells (41).

Tamoxifen, the most commonly used anti-cancer drug in the world (37), has estrogenic and antiestrogenic activities, depending on the target tissue. For instance, it is estrogenic on the uterine epithelium (where it is correlated with endometrial cancer) and bones (where it helps prevent osteoporosis), but antiestrogenic on the mammary epithelium (where it acts as a useful treatment option for breast cancer) (42).

SERMs are generally well tolerated, orally administered medications, but they are not devoid of adverse effects (43). Adverse events (AEs) include vaginal dryness and bleeding, hot flashes, sleep disturbances, weight gain, mood swings, depression, increased risk of endometrial cancer, and increased risk of thromboembolic events (43, 44). The likelihood of occurrence of these side effects increases with the duration of administration especially when these drugs are used for more than 12 months; nevertheless, it decreases with age (45).

Multiple randomized and non-randomized clinical trials in which patients with both advanced and early stages of breast cancer were treated with SERMs have shown efficacy, safety, and good tolerability of both tamoxifen and toremifene (46). For example, Chi et al. analyzed 23 trials with a total of 7,242 patients with advanced breast cancer (ABC). They found that toremifene is at least as effective as tamoxifen and could be an alternative to tamoxifen in ABC, even though it was associated with more AEs such as vaginal bleeding [odds ratio [OR] 0.45; 95% confidence interval [CI] 0.26–0.80; *p* < 0.05 (47).

Resistance to SERMs can either be *de novo* (48) or acquired (49). Multiple possible causes for resistance have been hypothesized, such as loss of ER expression and function (repression of receptor gene transcription after epigenetic modifications) (50), ER gene mutation, abnormal splicing (51), or possibly overpopulation of ER-negative cells with a heterogeneous ER-positive tumor (52). Another possible cause is abnormal expression of co-regulatory proteins, such as the one known as “amplified in breast cancer 1” (AIB1), which is usually overexpressed in resistant breast tumors (53). There are also some other pharmacological reasons, such as decreased influx or increased efflux of the drug, leading to decreased intracellular availability (54).

## Selective Estrogen Receptor Degrader: Fulvestrant

The estrogenic property of tamoxifen and the risk of developing resistance to this drug over the course of treatment have led to the development of newer therapeutic agents with different modes of action. Fulvestrant, a selective ER degrader, was introduced in 2002 as a second-line therapy for postmenopausal women with hormone-dependent ABC. In contrast to tamoxifen, fulvestrant does not carry an agonist effect in uterine tissue because it inhibits both AF1 and AF2. As a 7α-alkylsulphinyl analog of 17β-estradiol, it binds the ER competitively with a higher affinity (89% of that of estradiol) (55), antagonizing the activity of estradiol. Once fulvestrant binds to the ER, receptor dimerization is inhibited and energy-dependent nucleo-cytoplasmic shuttling is disrupted, thereby blocking localization of the receptor to the nucleus (56, 57).

Fulvestrant does not exhibit cross-resistance with tamoxifen. In other words, female patients who are resistant to tamoxifen may respond to treatment with fulvestrant (58), an aspect that has been recently investigated with the analysis of probable culprit inherent and acquired mutations in ESR1 (59, 60). Common AEs associated with fulvestrant include hot flashes and menopause-like symptoms (61).

Other more potent SERDs or SERM/SERD combinations are being studied in early models, especially in regards to clinical activity and benefit in presence of ESR1 mutations, such as pipendoxifene, bazedoxifene (62), AZD9496, GDC-0810 (63), ARN810, and RAD1901.

### CONFIRM Trial

To determine the optimal dosing of fulvestrant, the phase III CONFIRM trial (Comparison of Faslodex in Recurrent or Metastatic Breast Cancer) randomly assigned postmenopausal ER-positive patients with ABC to receive fulvestrant at a 250 mg dose vs. a 500 mg dose. The primary endpoint of the study was progression-free survival (PFS), which was significantly greater for fulvestrant 500 mg (hazard ratio [HR] 0.80; 95% CI 0.68–0.94; *p* = 0.006). Furthermore, the median OS for fulvestrant 500 mg was 26.4 vs. 22.3 months for fulvestranat 250 mg ([HR] 0.81; 95% CI 0.69–0.96; *p* = 0.02). The 500 mg dose of fulvestrant was associated with a 19% lower risk of death, with no difference in serious AEs when compared to lower dosing. Thus, the 500 mg dose of fulvestrant proved to be superior and became the standard of care (29, 64).

### Tamoxifen vs. Fulvestrant

Fulvestrant 250 mg was compared with tamoxifen in terms of time to progression, objective response rate, clinical benefit rate, time to treatment failure, time to death, and quality of life in a study of 581 postmenopausal women with ER-positive and/or PR-positive ABC who were endocrine therapy naïve or last had endocrine therapy at least 12 months before the study. There was a similar time to progression (6.8 months in the fulvestrant group vs. 8.3 months in the tamoxifen group; HR 1.18; 95% CI 0.98–1.44; *p* = 0.088). There were no significant differences in the other outcomes. AEs common to both groups were nausea, asthenia, vasodilation, and pain especially of bone. The trial results indicated that fulvestrant was similar in efficacy to tamoxifen, with no superiority or inferiority (30). Nevertheless, it is important to keep in mind that the dose of fulvestrant used in this trial was 250 mg, whereas 500 mg is currently the standard of care.

## Aromatase Inhibitors

Aromatase inhibitors are the next class of anti-estrogenic drugs to be discussed. They were first used as ovulation inducers in infertility clinics in the early 1990s (65). It was not until the late 1990s and early 2000s that aromatase inhibitors took their rightful place in the battle against breast cancer (66).

Aromatase is a cytochrome P450 enzyme involved in the synthesis of estrogen from androgens. After menopause, fat and muscle aromatase is responsible for circulating estrogen levels. In the breast, uterus, and other estrogen-sensitive tissues, aromatase provides local estrogen (67). Increased levels of aromatase in the mammary epithelium are associated with elevated tumor estrogen levels in postmenopausal women (68). Therefore, uncontrolled levels of aromatase promote tumor proliferation (69). Aromatase inhibitors (AIs) are used to block the activity of aromatase, thereby inhibiting estrogen biosynthesis. First- and second-generation AIs were removed from the market because of their numerous AEs, and they were replaced by third-generation AIs. There are two classes of third-generation AIs: steroidal AIs (SAIs) (exemestane) and non-steroidal AIs (NSAIs) (letrozole and anastrozole); the NSAIs bind competitively to the substrate binding site (70), whereas SAIs irreversibly bind to the active site of the aromatase enzyme and block substrate binding (70). High levels of the latter are required to effectively block the enzyme. A drawback of SAIs is that they may produce androgenic effects and reduce sex hormone-binding globulin levels in plasma (71). All AIs are administered in daily oral doses (72). Treatment is often accompanied with hot flushes, vaginal dryness, decreased libido and, fatigue, as well as joint and bone complications (osteoporosis) (73).

Some HR-positive/HER2-negative ABCs may be resistant to AI therapy. Wang et al. found that resistance to AI may be at the genetic and molecular levels, involving aromatase gene polymorphisms (74). Moreover, resistance was found more in patients with increased hypoxia-inducible factor-1 alpha and P44/42 MAPK (75), as well as low-molecular-weight cyclin E overexpression (76).

### Phase III Study of Letrozole vs. Tamoxifen as First-Line Treatment

Mourisden et al. compared a NSAI (letrozole) with tamoxifen as first-line treatment in postmenopausal women with HR-positive, treatment-naïve ABC. Median time to progression was improved in the letrozole arm vs. the tamoxifen arm (9.4 vs. 6.0 months, respectively; *p* < 0.0001), and overall survival and all other outcomes favored the letrozole arm, such as treatment failure, overall objective response rate and overall clinical benefit (31, 77). Furthermore, the total duration of endocrine therapy was significantly longer for participants receiving letrozole (*p* = 0.005), as was the time to worsening of performance status (*p* = 0.001) (31).

## Aromatase Inhibitors vs. Fulvestrant

### FALCON Trial

FALCON was the first phase III randomized, double-blind, multicenter trial to compare the efficacy and safety of monotherapy with fulvestrant 500 mg and a NSAI (anastrozole) in 462 endocrine therapy-naïve postmenopausal women with HR-positive ABC (32). PFS, the primary endpoint, was significantly higher in the fulvestrant group than in the anastrozole group (16.6 vs. 13.8 months, respectively; HR 0.797; 95% CI 0.637–0.999; *p* = 0.0486). The risk of progression in women with non-visceral disease was 41% lower in the fulvestrant group than in the anastrozole group (HR 0.592; 95% CI 0.419–0.837, mPFS 22.3 vs. 13.8 months, respectively). In patients with visceral disease, the treatment effects for fulvestrant and anastrozole were comparable (HR 0.993; 95% CI 0.740–1.331) (78). Health-related quality of life was equivalent with the two treatments. The most common AEs were arthralgia with fulvestrant and hot flushes with anastrozole. The results of this trial indicated that fulvestrant has greater efficacy and tolerability than anastrozole in treatment-naïve postmenopausal women with HR-positive metastatic breast cancer (32).

### EFECT Trial

EFECT on the other hand, was a multicenter, placebo-controlled phase III trial designed to compare fulvestrant and a SAI (exemestane) in postmenopausal women with HR-positive cancer; this study involved patients who had progressed or relapsed after previous NSAI therapy. A total of 693 women were randomly assigned to receive either fulvestrant (*n* = 351) at a loading dose of 500 mg followed by 250 mg on days 14 and 28 then every 28 days, or exemestane (*n* = 342). The two groups had a median time to progression (primary endpoint) of 3.7 months (HR 0.963; 95% CI 0.819–1.133; *p* = 0.6531). Thirty-eight participants were examined for the clinical benefit rate of each drug, with fulvestrant (*n* = 20) having a 32.2% clinical benefit rate, vs. 31.5% for exemestane (*n* = 18) (OR 1.03; 95% CI 0.71–2.487; *p* = 0.853). No significant difference was found between the two drugs in terms of time to progression and clinical benefit rate, indicating that they have equal efficacy and tolerability in postmenopausal women with HR-positive ABC (33).

## Monotherapy vs. Combination Therapy

Three trials have been conducted to compare monotherapy and combination therapy. The FACT trial demonstrated that the combination of a selective ER degrader and an AI was not superior to an AI alone. However, the SWOG trial thereafter did show superiority. Moreover, the SoFEA trial substantiated the results of the initial FACT trial, showing no real difference between combination therapy and monotherapy.

### FACT Trial

A total of 514 postmenopausal or premenopausal women receiving a gonadotropin-releasing hormone (GRH) agonist, who had HR-positive disease that progressed after primary treatment, were randomly assigned to receive IM fulvestrant 500 mg and an additional monthly injection (250 mg) plus anastrozole 1 mg daily (experimental arm) or anastrozole 1 mg daily alone (standard arm).

The primary endpoint, time to progression, was found to be 10.8 and 10.2 months in the experimental and standard arms, respectively (HR 0.99; 95% CI 0.81–1.20; *p* = 0.91); median overall survival, one of the secondary endpoints, was 37.8 and 38.2 months, respectively (HR 1.0; 95% CI 0.76–1.32; *p* = 1.00). These findings indicated that in this trial, fulvestrant combined with anastrozole was not superior to anastrozole alone in the treatment of ABC. Both groups manifested similar AEs, with hot flashes being more common in the experimental arm; death from AEs was reported in 11 patients in the experimental arm vs. five patients in the standard arm (34).

### SWOG Trial

A total of 694 postmenopausal women with previously untreated ABC were randomly assigned, in a 1:1 ratio, to receive either 1 mg anastrozole orally daily (group 1), with crossover to fulvestrant alone strongly encouraged if the disease progressed, or anastrozole and fulvestrant in combination (group 2). Patients were stratified according to prior or no prior receipt of adjuvant tamoxifen therapy. Fulvestrant was administered at a dose of 500 mg on day 1 and 250 mg on days 14 and 28 then monthly thereafter. The primary endpoint was PFS, with overall survival (OS) designated as a prespecified secondary outcome.

The median PFS was 13.5 months in group 1 and 15.0 months in group 2 (HR 0.80; 95% CI 0.68–0.94; *p* = 0.007). The combination therapy was generally more effective than anastrozole alone in all subgroups, with no significant interactions. OS was also longer with combination therapy (median 41.3 months in group 1 and 47.7 months in group 2; HR 0.81; 95% CI 0.65–1.00; *p* = 0.05), despite the fact that 41% of the patients in group 1 crossed over to fulvestrant after progression. Three treatment-related deaths were noted in group 2, but rates of grade 3–4 toxicities did not differ significantly. The combination of anastrozole and fulvestrant was superior to anastrozole alone or sequential anastrozole and fulvestrant for the treatment of HR-positive ABC, despite the use of a dose of fulvestrant that was below the cu6rrent standard (35)

### SoFEA Trial

In this composite multicenter phase III trial, 723 postmenopausal women with HR-positive/HER2-negative ABC progressing after NSAI treatment were randomly assigned to receive fulvestrant (500 mg loading, followed by 250 mg IM on days 15 and 29 and every 28 days thereafter) plus anastrozole (1 mg orally once daily) (*n* = 243), fulvestrant plus anastrozole-matched placebo (*n* = 231) or exemestane (25 mg orally once daily) (*n* = 249).

PFS, the primary endpoint, was found to be 4.4 months (95% CI 3.4–5.4) in the fulvestrant plus anastrozole group, 4.8 months (95% CI 3.6–5.5) in the fulvestrant plus placebo group, and 3.4 months (95% CI 3.0–4.6) in the exemestane group. There were no significant differences between the first 2 groups (HR 1.00, 95% CI 0.83–1.21; log-rank *p* = 0.98) and the second 2 groups (HR 0.95, 95% CI 0.79–1.14; log-rank *p* = 0.56). The AEs common to the 3 groups were arthralgia, lethargy, nausea, and vomiting (36).

This trial demonstrates the lack of superiority of the combination of a selective ER degrader and anastrozole compared with fulvestrant alone for women with HR-positive ABC who have relapsed after previous NSAI treatment. Likewise, it confirms the results of the EFECT trial, indicating that there is no significant difference between fulvestrant and exemestane treatment in this setting.

## Overcoming Resistance to Endocrine Therapy

One of the main challenges in the treatment of HR-positive ABC is to bypass resistance to endocrine therapy. This form of cancer is usually treated with multiple lines of endocrine treatment until resistance or non-responsiveness is noted. Patients can experience resistance during exposure to all of the previously mentioned drug classes. There are different mechanisms of resistance: it can arise *de novo* or be acquired via up-regulation of certain alternative signaling pathways.

Multiple studies were performed to try to uncover the potential mechanisms of endocrine resistance, including pathways that interact with ER, as well as its co-regulators or transcriptional factors, ultimately altering ER activity, ER sensitivity to endocrine treatments with decreased ER binding within the ER cristome (79), and/or possibly leading to escape pathways that allow cancer cells to bypass ER dependency via alternative proliferative stimuli sustaining tumor growth and disease progression. There remain other etiologic hypotheses of resistance, such as tumor heterogeneity, over-population of ER-negative cells, as well as clonal selection of estrogen independent mutant cells, and pharmacologic capacity of resistant cells to efflux drugs.

The most investigated mechanism of resistance is through affecting ER constitutive ligand-independent transcriptional activity, driven by genomic, epigenetic, and tumor microenvironment influences. Retrospective studies discussed the role of ESR1 and ligand binding domain (LBD) mutations leading to such resistance with different mutations leading to different outcomes. These mutations are associated with prognostic as well as predictive characteristics, and mutant tumors are considered more aggressive with poorer prognosis, and more propensity to metastasize with less responsiveness to endocrine therapy (80) (complete resistance to AI, lower activity with SERMs/SERDs), as shown by secondary analysis from BOLERO-2, PALOMA-3 and SOFEA trials.

Mutant ER recruits coactivators in the absence of hormone, and alters the conformational dynamics of the loop connecting Helix 11 and Helix 12 in the LBD of ER that leads to a stabilized agonist state and an altered antagonist state that resists inhibition with decreased affinity to estradiol, tamoxifen and fulvestrant (81, 82).

Zhang et al. were the first to describe ESR1 mutations in 1997, and since then, multiple resistance-culprit LBD mutations have been recognized and studied, not only in tumor tissue but also in circulating tumor cells (CTCs) and circulating tumor DNA (CtDNA) using newer more sensitive techniques such as the digital PCR (dPCR) or droplet digital PCR (ddPCR) (80). To note, mutations were more likely to be found in plasma samples of patients with metastatic disease rather than tissue and more rarely in primary tumor tissue (83, 84), suggesting the possibility of acquired mutation leading to resistance or selection of cells with mutation while on adjuvant hormonal therapy, with some studies taking serial mutational analysis showing increase in allele frequency with treatment.

The most common detected ESR1 mutations are D538G (most common- 36%), Y537S (14%), L536Q, Y537N, Y537C, S463P, and E380QE. Toy et al. studied these different mutations and their different effect on ER activation. What they found was that although all mutations caused resistance to AIs, only selected mutants such as Y537S caused significant changes associated with fulvestrant resistance *in vitro*. However, more potent SERDs with higher bio-availability such as AZD9496 inhibited tumors driven by Y537S more effectively than fulvestrant, whereas the inhibition was equivalent in tumors driven by D538G, E380Q, or S463P (63).

Moreover, other possible culprit mutations and pathways of resistance were also investigated with a notable role of KRAS mutations (85), and a major role of PI3K/AKT pathway, such as PIK3CA mutations that can occur in up to 40% of ER-positive, HER2- negative breast tumors, as well as RB1, ERBB2, FGFR2/3 mutations, and others.

In an effort to overcome resistance, researchers have tested various drugs that might overcome resistant mutations and target key intracellular signaling pathways in order to improve disease outcome. Options are expanding with the introduction of targeted agents such as inhibitors of cyclin-dependent kinases 4 and 6 (CDK 4/6), inhibitors of mammalian target of rapamycin (mTOR), inhibitors of PI3K/AKT pathways, histone deacetylase inhibitors, new oral SERDs, new SERM/SERD combinations and others (Table 2).

**Table 2:** Overcoming resistance to endocrine therapy.

**Trial/ Phase**	**Intervention Comparison**	**T#**	**# pt**	**Pt characteristics**			**Disease characteristics**		**Previous therapy**		**Surviva**l			
				**Age**	**Menopausal status**									
					**Pre meno**	**Post meno**	**HR +ve**	**HER2 –ve**	**Chemo**	**Endocrine**	**OS**	**PFS or TTP**	**CBR**	**ORR**
**INHIBITORS OF CYCLIN-DEPENDENT KINASES 4 AND 6**														
Finn et al. (86)	Palbociclib + letrozole	1	444	62	0	100	100	100	48	56.1	NR	24.8	84.9	42.1
Phase 3	Placebo + letrozole		222	61	0	100	100	100	49.1	56.8	NR	14.5	70.3	34.7
	*p*											<0.001	<0.001	0.06
Cristofanilli et al. (87)	Fulvestrant + palbociclib	=>2	347	57	21	79	100	100	40	100	34.9	9.5	67	19
Phase 3	Fulvestrant + placebo		174	56	21	79	100	100	43	100	28	4.6	40	9
	*p*										0.09	<0.0001	<0.0001	0.0019
Hortobagyi et al. (88)	Ribociclib plus letrozole	1	334	62	0	100	100	100	43.7	52.4	NR	25.3	79.9	42.5
Phase 3	placebo plus letrozole		334	63	0	100	100	100	43.4	51.2	33	16	73.1	28.7
	*p*											9.63 × 10^−8^	NS	9.18 × 10^−5^
Slamon et al. (89)	Ribociclib + Fulvestrant	=>1	484	63	0	100	100	100	A43.2,N13.4∧	48.8	NR	20.5	70.2	32.4
Phase 3	Placebo + fulvestrant		242	63	0	100	100	100	A41.7,N12.4∧	45	NR	12.8	62.8	21.5
	*p*											<0.001	0.02	<0.001
Tripathy et al. (90) Phase 3	Ribociclib + ET	=>1	335	43	100	0	100	100	55	38	NR	23.8	79	41
	Placebo + ET		337	45	100	0	100	100	55	42	NR	13	70	30
	*p*											<0.0001	0.0020	0.00098
Sledge et al. (91)	Abemaciclib + fulvestrant	2	446	59	16.1	83.2	100	100	59.9	100	NR	16.3	72.2	35.2
Phase 3	Placebo + fulvestrant		223	62	18.8	80.7	100	100	60.1	100	NR	9.3	56.1	16.1
	*p*											<0.001	<0.001	<0.001
Goetz et al. (92)	Abemaciclib + NSAI	1	328	63	0	100	100	100	38.1	45.7	NR	NR	78	48.2
Phase 3	Placebo + NSAI		165	63	0	100	100	100	40	49.5	NR	14.7	71.5	34.5
	*p*											0.000021	0.101	0.002
**INHIBITORS OF MAMMALIAN TARGET OF RAPAMYCIN**														
Yardley et al. (93)	Everolimus + exemestane	2	485	62	0	100	100	100	69	84	31	7.8	51.3	12.6
Phase 3	Exemestane + placebo		239	61	0	100	100	100	65	84	26.6	3.2	26.4	1.7
	*p*										0.1426	<0.0001	<0.0001	<0.0001
Wolff et al. (94)	Letrozole + temsirolimus	1	555	63	0	100	96	40	65	43	NR	8.9	NR	27
Phase 3	Letrozole + placebo		555	63	0	100	95	47	59	40	NR	9	NR	27
	*p*											0.25		NS
Bachelot et al. (95)	Tamoxifen + everolimus	2	54	63	0	100	100	98	70	100	NR	TTP, 8.6	61	NR
Phase 2	Tamoxifen		57	66	0	100	100	93	82	100	32.9	4.5	42	NR
	*p*										<0.05	<0.05	<0.05	
**PI3K INHIBITORS**														
Baselga et al. (96)	Buparlisib + fulvestrant	=>2	576	62	0	100	100	100	24	100	33.2	6.9	43.8	11.8
Phase 3	Placebo + fulvestrant		571	61	0	100	100	100	31	99	30.4	5	42	7.7
	*p*										NS	0.00021	NS	NS
Baselga et al. (97)	Buparlisib plus fulvestrant	=>2	289	60	0	100	100	100	36	100	NR	3.9	71	8
Phase 3	Placebo plus fulvestrant		143	62	0	100	100	100	35	100	NR	1.8	22	2
	*p*											0.0003	NS	NS
Baselga et al. (98)	Taselisib + fulvestrant	=>2	417	60	0	100	100	100	NR	100	26.8	7.4	51.5	28
Phase 3	Placebo + fulvestrant		214	61	0	100	100	100	NR	100	23.6	5.4	37.3	11.9
	*p*										0.85	NS		0.0002
André et al. (99)	Alpelisib + fulvestrant	=>2	NR	NR	0	100	100	100	NR	100	NR	11	NR	36
Phase 3	Placebo + fulvestrant		NR	NR	0	100	100	100	NR	100	NR	5.7	NR	16
	*p*											0.00065		0.0002
**HISTONE DEACYTELASE INHIBITORS**														
Yardley et al. (100)	Exemestane + entinostat	2	64	63	0	100	98	92	58	100	28.1	4.28	28.1	6.3
Phase 2	Exemestane + placebo		66	62	0	100	98	89	67	100	19.8	2.27	25.8	4.6
	*p*										0.036	0.055	0.78	0.58
Jiang et al. (101)	Chidamide + exemestane	2	244	NR	0	100	100	100	NR	100	NR	7.4	46.7	18.4
Phase 3	placebo + exemestane		122	NR	0	100	100	100	NR	100	NR	3.8	35.5	9.1
	*p*											0.0336	0.034	0.026
**ANTI-ANGIOGENIC AGENTS**														
Gray et al. (102)	paclitaxel + bevacizumab	1	368	56	NR	NR	60.6	92.5	66.3	90.8	26.7	11.3	NR	48.9
Phase 3	paclitaxel		354	55	NR	NR	63	89.9	65.3	89.3	25.2	5.9	NR	22.2
	*p*										0.16	<0.0001		<0.0001
Pivot et al. (103) Phase 3	docetaxel + bevacizumab_7.5_	1	248	54	NR	NR	78	100	65	85	30.8	9	NR	55.2
	docetaxel + bevacizumab_15_		247	55	NR	NR	76	100	68	77	30.2	10.1	NR	64.1
	docetaxel + placebo		241	55	NR	NR	78	100	65	88	31.9	8.2	NR	46.4
	*p*										NS	0.12,0.006		0.07,0.001
Martin et al. (104)	ET + bevacizumab_15_	1	190	64	0	100	100	100	43.7	52.6	52.1	19.3	76.8	40.8
Phase 3	ET		184	66	0	100	100	100	47.8	51.6	51.8	14.4	67.4	21.9
	*p*										0.518	0.126	0.041	<0.001
**PROTEASOME INHIBITORS**														
Adelson et al. (105)	Fulvestrant alone	2	59	57	0	100	100	100	27	66^*^	NR	2.69	NR	NR
Phase 2	Fulvestrant + bortezomib		57	59	0	100	100	100	30	30^*^	NR	2.73	NR	NR
	*p*											0.06		

T#, Treatment line; # pt, Number of patients; OS, Overall survival; PFS, Progression free survival; TTP, Time to progression; CBR, Clinical benefit rate; ORR, Objective response rate; NR, Not reported; NS, Not significant;

* *Rate of Endocrine therapy used for metastatic disease; ^A^, Adjuvant; N, Neoadjuvant*.

## Inhibitors of Cyclin-Dependent Kinases 4 and 6

The cell cycle involves various regulators that allow the cell to grow and proliferate. Cyclin-dependent kinases (CDKs) are examples of such regulators. They bind cyclins, which trigger the cell cycle to proceed through the checkpoints (G1 to S and G2 to M). Control over the cell cycle can be disrupted by various factors, including oncogenes (gain of function) and tumor suppressor genes (loss of function), causing non-stop activation of CDKs. Retinoblastoma (RB) gene product binds to E2F to inhibit the cell cycle. Once CDK4 and 6 are bound to cyclin D because of extracellular signals (106), RB is phosphorylated, then it detaches from E2F and allows the cell cycle to proceed from G1 to S. As a result, CDKs are targets for anti-cancer therapy (107).

One of the first CDK inhibitors was alvocidib. It is a pan-CDK inhibitor that induces apoptosis, suppresses transcription, and stimulates autophagy; however, it is very non-selective and is associated with many toxicities (neutropenia, hyperglycemia, cardiac, and pulmonary dysfunction). In contrast, palbociclib is an oral CDK 4/6 inhibitor that prevents the cell cycle to proceed from G1 to S phase, thus inhibiting cell proliferation. It was the first in its class to be approved for cancer treatment, followed shortly thereafter by ribociclib and abemaciclib.

### PALOMA-2 Trial

PALOMA-2 was a phase III trial that followed PALOMA-1, a phase II study that demonstrated a prolonged PFS with palbociclib plus letrozole compared with letrozole alone in postmenopausal women with treatment-naïve HR-positive/HER2-negative ABC.

In PALOMA-2, 666 postmenopausal women were randomly assigned in a ratio of 2:1 to receive palbociclib 125 mg daily on days 1–21 of 28 days cycles plus letrozole 2.5 mg daily vs. placebo plus letrozole 2.5 mg daily. The median PFS, as the primary endpoint, was 24.8 months in the palbociclib arm vs. 14.5 months in the placebo arm (HR 0.58; 95% CI 0.46–0.72; *p* < 0.001).

This trial showed that previously untreated postmenopausal women with HR-positive/HER2-negative breast cancer would respond better to palbociclib plus letrozole than to letrozole alone in terms of PFS. The most common adverse effects were neutropenia, leukopenia, anemia, and fatigue (86).

### PALOMA-3 Trial

PALOMA-3 was a double-blind, placebo-controlled, multicenter phase III trial involving 521 women aged 18 years and older with HR-positive/HER2-negative ABC who progressed or relapsed within a year after receiving endocrine therapy. They were randomly assigned to receive either oral palbociclib 125 mg daily for 3 weeks followed by 1 week off and fulvestrant 500 mg IM on days 1, 15, and 28 and then every 28 days thereafter (group 1) or fulvestrant at the same dose along with placebo for 3 weeks followed by 1 week off (group 2). The purpose was to assess the efficacy and safety of palbociclib administered in combination with fulvestrant, compared with fulvestrant alone (87).

The median PFS in group 1 was 9.5 months (95% CI 9.2–11.0) vs. 4.6 months (95% CI 3.5–5.6) in group 2 (HR 0.46; 95% CI 0.36–0.59, *p* < 0.0001). The median OS in group 1 was 34.9 months (95% CI 28.8–40.0) vs. 28.0 months in group 2 (95% CI 23.6–34.6) (HR 0.81; 95% CI 0.64–1.03; *p* = 0.09; absolute difference, 6.9 months).

Among 410 patients with sensitivity to previous endocrine therapy, the median OS was 39.7 months (95% CI 34.8–45.7) in the palbociclib-fulvestrant group and 29.7 months (95% CI 23.8–37.9) in the placebo-fulvestrant group (HR 0.72; 95% CI 0.55–0.94; absolute difference 10 months).

The median time to chemotherapy was 17.6 months in the palbociclib-fulvestrant group vs. 8.8 months in the placebo-fulvestrant group (HR 0.58; 95% CI 0.47–0.73; *p* < 0.001).

The most common AEs experienced in both groups were neutropenia, anemia, and leukopenia (87).

This trial demonstrated that palbociclib in combination with fulvestrant, is useful for treatment-naïve and endocrine therapy-resistant patients, with improvement in PFS, a numerical increase in OS and doubling of time to chemotherapy.

### MONALEESA-2 Trial

In this randomized controlled phase III trial, postmenopausal women with treatment-naïve HR-positive/HER2-negative ABC were randomly assigned to receive letrozole (2.5 mg/day, continuous) with ribociclib (600 mg/day, 3 weeks on/1 week off) (group 1, *n* = 334), vs. letrozole with placebo (group 2, *n* = 334). The primary endpoint was PFS, median PFS was prolonged by 9.3 months, from 16.0 months (95% CI 13.4–18.2) for patients receiving placebo plus letrozole to 25.3 months (95% CI 23.0–30.3) for those receiving ribociclib plus letrozole. The overall response rate (ORR) to ribociclib plus letrozole was higher than that to letrozole alone (42.5 vs. 28.7%; *p* = 9.18 × 10^−5^). After a median duration of follow-up of 26.4 months, median overall survival was not reached in the treatment group at time of second interim analysis compared to 33 months in the placebo group. As for toxicities, 76.9% of patients in the first group experienced neutropenia (as well as a higher rate of leukopenia), compared with 5.8% of patients in the second group.

As a first-line treatment combination, ribociclib and letrozole together has shown to increase PFS in postmenopausal women with HR-positive/HER2-negative ABC (88, 108).

### MONALEESA-3 Trial

MONALEESA-3 was another randomized phase III study that evaluated ribociclib, but this time it was used in combination with fulvestrant in 726 postmenopausal women with HR-positive/HER2-negative ABC who were treatment naïve or had received up to 1 line of prior endocrine therapy. Study participants were randomly assigned at a 2:1 ratio to receive either ribociclib plus fulvestrant or placebo plus fulvestrant.

Median PFS (the primary endpoint) was significantly improved with ribociclib plus fulvestrant vs. placebo plus fulvestrant: 20.5 months (95% CI 18.5–23.5) vs. 12.8 months (95% CI 10.9–16.3), respectively (HR 0.593; 95% CI 0.480–0.732; *p* < 0.001).

A similar treatment effect was noted between patients who were treatment naïve in the advanced setting and patients who had received 1 prior line of endocrine therapy for advanced disease. For measurable disease, the overall response rate was 40.9% for the ribociclib plus fulvestrant arm and 28.7% for the placebo plus fulvestrant arm. Neutropenia was the most significant AE (89).

### MONALEESA-7 Trial

Although this article stresses on postmenopausal women given that most advanced breast cancer trials included postmenopausal women, and treatment of premenopausal women was extrapolated from those same trials as they received proper ovarian function suppression and rendered postmenopausal. Nevertheless, it is important to mention Monaleesa 7, the first phase III trial to study premenopausal ladies, designed specifically to assess the efficacy and safety of ribociclib plus endocrine therapy in 672 premenopausal women with advanced, HR-positive/HER2- negative breast cancer, who had not received previous treatment with cyclin-dependent kinases 4 and 6 inhibitors but who may have received endocrine therapy and chemotherapy in the adjuvant or neoadjuvant setting as well as up to 1 line of chemotherapy for advanced disease. Patients were randomly assigned to receive either oral ribociclib (600 mg/day on a 3-weeks-on, 1-week-off schedule) or matching placebo with either oral tamoxifen (20 mg daily) or a NSAI (letrozole 2.5 mg or anastrozole 1 mg, both oral, daily), all with goserelin (3.6 mg administered subcutaneously on day 1 of every 28 days cycle).

The median PFS in the ribociclib group was significantly higher (23.8 months [95% CI 19.2–NR] vs. 13.0 months [95% CI 11.0–16.4]) (HR 0.55; 95% CI 0.44–0.69; *p* < 0.0001). Grade 3 or 4 AEs, which were mostly neutropenia and leukopenia, were significantly higher in the ribociclib group with no treatment-related deaths occurring (90).

### MONARCH 2 and 3 Trials

In MONARCH 2, a global, double-blind, phase III study, 669 postmenopausal women with HR-positive/HER2-negative ABC progressing on or ≤12 months from their last endocrine therapy were randomly assigned 2:1 to receive abemaciclib or placebo (150 mg twice daily) on a continuous schedule and fulvestrant (500 mg, per label). The trial compared the efficacy and safety of abemaciclib, a selective cyclin-dependent kinase 4 and 6 inhibitor, plus fulvestrant vs. fulvestrant alone in patients with ABC.

Abemaciclib plus fulvestrant significantly improved PFS (the primary endpoint) compared with fulvestrant alone (median, 16.4 vs. 9.3 months; HR 0.553; 95% CI 0.449–0.681; *p* < 0.001). In patients with measurable disease, abemaciclib plus fulvestrant achieved an ORR of 48.1% (95% CI 42.6–53.6) compared with 21.3% (95% CI 15.1–27.6). The most common AEs in the abemaciclib vs. placebo arms were diarrhea (86.4 vs. 24.7%), neutropenia (46.0 vs. 4.0%), nausea (45.1 vs. 22.9%), and fatigue (39.9 vs. 26.9%) (91).

MONARCH 3, on the other hand, which was also a double-blind, randomized, phase III study, assessed abemaciclib combined with an AI (letrozole or anastrozole) in postmenopausal women with treatment-naïve HR-positive/HER2-negative ABC. In this study, 493 women were randomly assigned to receive 150 mg of abemaciclib (group 1) or placebo (group 2) orally twice daily, in combination with either 2.5 mg of letrozole or 1 mg of anastrozole daily.

Median PFS, the primary endpoint, was significantly prolonged in the abemaciclib arm compared with the placebo arm (NR vs. 14.7 months, respectively, HR 0.54; 95% CI 0.41–0.72; *p* = 0.000021). In measurable disease, the ORR was also higher in the abemaciclib arm than in the placebo arm (59 vs. 44%) (*p* = 0.004). Grade 1 diarrhea as well as neutropenia and leukopenia were the most frequent AEs, being more frequent in the abemaciclib arm (92).

Abemaciclib has been shown to be associated with a higher monotherapy response rate than other CDK 4/6 inhibitors. This may be attributed to its continuous dosing schedule as well as its higher potency for CDK 4 inhibition, which ultimately leads to tumor regression (109), as well as its recently noted role in activating the immune response in tumor microenvironment (110, 111).

Abemaciclib is associated with a significantly lower risk of neutropenia than palbociclib and ribociclib (112) because of its higher selectivity for CDK4/cyclin D1considering that inhibition of CDK6 affects hematopoiesis and circulating neutrophils, in addition to absence of effect on other CDKS such as CDK9 responsible for apoptosis (113). Moreover, recent reports suggest better penetration of the central nervous system with abemaciclib (114).

### PALOMA-4 Trial

PALOMA-4 (NCT02297438) is an ongoing phase III trial comparing the clinical benefit of treatment with letrozole (2.5 mg, orally once daily, continuously) and palbociclib (125 mg, orally once daily on days 1 to 21 of every 28 days cycle, followed by 7 days off treatment) with that of letrozole alone in the first-line treatment of 339 Asian postmenopausal women with HR-positive/HER2-negative ABC. The primary outcome is PFS; secondary endpoints include OS, ORR, duration of response and quality of life. The study is currently active. It is no longer recruiting and is expected to be completed in November 2019^1^^,^^2^^,^^3^.

## Inhibitors of Mammalian Target of Rapamycin/Inhibitors of the PI3K Pathway

The PI3K/AKT/mTOR signaling pathway is crucial for maintaining control of proliferation in mammalian cells (115), where uncontrolled activation results in unstoppable proliferation of tumor cells in cancers.

Receptor tyrosine kinases (RTKs) activate a subgroup of the PI3K family, which is class I_A_ PI3Ks. This subgroup acts to convert phosphatidylinositol 4,5-bisphosphate (PIP_2_) to phosphatidylinositol 3,4,5-triphosphate (PIP_3_). The latter activates AKT, which regulates cell growth and proliferation and activates mammalian target of rapamycin (mTOR) (116).

mTOR, also known as FRAP (FKBP12-rapamycin-associated protein), is a serine threonine kinase that regulates cell proliferation. Its signaling pathway is controlled by growth factors, nutrients, amino acids, plus energy and stress signals including ATP and O_2_ levels. These allow it to activate downstream mechanisms including cell division, transcription, and translation (117). mTOR comprises two protein complexes: mTORC1 and mTORC2 (118, 119). mTORC1's major role is to regulate protein synthesis. It is activated by the phosphatidylinositol 3-kinase (PI3K)/AKT pathway. mTORC2 is activated by growth factors and is involved in cell survival and migration. It also controls the actin cytoskeleton (120).

One of the important mechanisms of resistance to endocrine therapy in HR-positive breast cancer is anomalous signaling through the PI3K/AKT mTOR pathway (121–124). The ER and mTOR signaling pathways are related in the following way: S6 kinase 1, a substrate of mTORC1, phosphorylates the activation function domain 1 or the ER responsible for ligand-independent receptor activation (125, 126).

Everolimus and temsirolimus inhibit the intracellular protein FKB12, which interacts with mTORC1, inhibiting mTOR signaling (127, 128); thereby representing a possible therapeutic option.

Other drugs targeting mutations associated with resistance have been studies, including PIK3CA mutations and PI3K inhibitors such as buparlisib (pan-PI3K inhibitor), taselisib, alpelisib, and pictilisib (98, 129).

## m-TOR Inhibitors

### TAMRAD Trial

In a phase II trial involving postmenopausal women with HR-positive/HER2-negative ABC, 111 participants were randomly assigned into 2 groups: the first received tamoxifen (20 mg/day) (*n* = 57), and the second received tamoxifen plus everolimus (10 mg/day) (*n* = 54) (95, 130).

The primary endpoint was clinical benefit rate, and it was found to be 42.1% (95% CI 29.1–55.9) in the tamoxifen group and 61.1% (95% CI 46.9–74.1) in the everolimus plus tamoxifen group.

Therefore, tamoxifen combined with everolimus proved to be superior to tamoxifen alone in this setting. There were no significant differences in the level of safety between the groups, and toxicity was tolerable. Grade 3–4 AEs included stomatitis and pain.

### BOLERO-2 Trial

In BOLERO-2, a phase III, double-blind trial investigating the role of everolimus by comparing everolimus plus exemestane vs. placebo plus exemestane in postmenopausal women with HR-positive/HER2-negative ABC and resistance to NSAIs (131), 724 women were divided into 2 groups in a 2:1 ratio. A total of 485 women received exemestane (25 mg/day) plus everolimus (10 mg/day), and 239 received exemestane (25 mg/day) plus placebo (80). Median PFS was 7.8 months for the first group and 3.2 months for the second group (HR 0.45; 95% CI 0.38–0.54); *p* < 0.0001).

The combination of a SAI with an inhibitor of signal transduction pathway yielded good results, prolonging PFS; however, it was not without toxicity. Serious AEs included death because of pneumonia, tumor hemorrhage, cerebrovascular events, renal failure, and suicide, in addition to stomatitis, anemia, fatigue, and pneumonitis (93, 131).

### HORIZON Trial

In this phase III randomized placebo-controlled study, 1,112 postmenopausal women with AI-naïve, HR-positive ABC were randomly assigned to receive either letrozole (2.5 mg orally daily) plus temsirolimus (30 mg daily for 5 days every 2 weeks), or letrozole plus placebo.

There was no significant difference in PFS (the primary endpoint) between the two arms (median 9 months; HR 0.90; 95% CI 0.76–1.07; *p* = 0.25). However, an unplanned exploratory sub-analysis showed improved PFS favoring letrozole/temsirolimus in patients ≤65 years of age (9.0 vs. 5.6 months; HR 0.75; 95% CI 0.60–0.93; *p* = 0.009).

Patients in the letrozole/temsirolimus arm experienced more grade 3 to 4 events (37 vs. 24%), including hyperglycemia, diarrhea, mucositis/stomatitis, and hyperlipidemia (94).

### BALLET Trial

Then came a European multicenter phase IIIb trial that included 2,131 Italian postmenopausal women with HR-positive/HER2-negative ABC who had progressed following prior endocrine therapy. They were stratified to those who had received prior chemotherapy (64%) and those who had not. All received everolimus plus exemestane in an attempt to evaluate the safety of such a regimen, specifically after chemotherapy.

Most patients discontinued treatment (97%) because of disease progression, financial issues, or AEs, with the most common AE being stomatitis attributed to everolimus. There was no difference in AEs between the 2 groups, with or without prior chemotherapy (132).

## PI3K Inhibitors

### BELLE-2 Trial

BELLE-2 was a phase III trial involving postmenopausal participants with HR-positive/HER2-negative ABC that had progressed after adjuvant AI therapy. A total of 1,147 participants were randomly assigned to receive fulvestrant (500 mg IM on days 1 and 15, then every 28 days thereafter) plus buparlisib (100 mg/day starting from day 15) or fulvestrant plus placebo. Median PFS was 6.9 months (95% CI 6.8–7.8) in the buparlisib group (*n* = 576), and 5 months (95% CI 4.0–5.2) in the placebo group (*n* = 571) (HR 0.78; 95% CI 0.67–0.89; *p* = 0.00021).

This indicated significant improvement in PFS when buparlisib was added to fulvestrant in HR-positive/HER2-negative AI-resistant ABC. Grade 3–4 AEs in the buparlisib group were increased aminotransferases, hyperglycemia, and rash (96).

### BELLE-3 Trial

In BELLE-3, a double-blind, placebo-controlled, phase III trial, 432 postmenopausal women aged 18 years and older with histologically confirmed HR-positive/HER2-negative ABC who had relapsed on or after endocrine therapy and treatment with mTOR inhibitors were randomly assigned by visceral disease status to receive oral buparlisib (100 mg per day) (*n* = 289) or matching placebo (*n* = 143) in addition to fulvestrant (500 mg) on days 1 and 15 of cycle 1 and on day 1 of subsequent 28 days cycles. The primary endpoint was PFS.

Median PFS was significantly longer in the buparlisib group than in the placebo group (3.9 months [95% CI 2.8–4.2] vs. 1.8 months [95% CI 1.5–2.8]; HR 0.67; 95% CI 0.53–0.84; *p* = 0.00030). AEs in the buparlisib vs. placebo group were elevated aminotransferases (22 vs. 3% for ALT, 18 vs. 3% for AST), hyperglycemia (12% vs. none), hypertension (6 vs. 4%), and fatigue (3 vs. 1%]), with serious AEs reported in 22% in the buparlisib group vs. 16% in the placebo group, mainly being elevated transaminases, dyspnea, and pleural effusion. On-treatment deaths occurred in 3% of patients in the buparlisib group and in 4% of patients in the placebo group, mostly because of disease progression. These findings indicated that buparlisib along with fulvestrant did improve PFS, but the safety profile did not support further development of the drug (97).

### SANDPIPER Trial

This double-blind, placebo-controlled, randomized, phase III trial tested the efficacy and safety of taselisib, a potent and selective PI3K inhibitor, when added to fulvestrant in postmenopausal patients with HR-positive/HER2-negative, *PIK3CA*-mutant ABC, recurring or progressing on AIs. A total of 631 postmenopausal patients were randomly assigned 2:1 to receive either taselisib (4 mg qd) or placebo in combination with fulvestrant.

The primary endpoint was investigator-assessed PFS. Other endpoints included OS, ORR, clinical benefit rate, duration of OR, safety, pharmacokinetics, and patient-reported outcomes. The group receiving taselisib had a 30% lower risk of progression, with time to progression extended by a median of 2 months (7.4 months with taselisib/fulvestrant vs. 5.4 months with fulvestrant/placebo). ORR doubled with the addition of taselisib (28 vs. 11.9%). Final OS data are not yet available.

AEs included diarrhea, hyperglycemia, and colitis, with 17% of patients in the taselisib group stopping treatment early.

The results of this trial suggest that taselisib is of some benefit. However, the benefits and risks ought to be weighed when the final results of the study are reported. The expected study completion date is July 2021 (98).

### SOLAR-1 Trial

SOLAR-1 is yet another international, randomized, double-blind, placebo-controlled, phase III trial evaluating the role of alpelisib, an alpha-specific PI3K inhibitor, plus fulvestrant in HR-positive/HER2- negative ABC in postmenopausal women and men with PIK3CA mutations progressing on or following treatment with an AI with or without CDK4/6 inhibitors.

In this study, 572 patients, 341 of whom had a PIK3CA-mutation, were randomly assigned 1:1 to receive continuous oral treatment with alpelisib 300 mg or placebo daily in combination with fulvestrant 500 mg IM injections on days 1 and 15 of the first cycle and day 1 of each subsequent 28 days cycle.

There was improvement in PFS for patients with a PIK3CA mutation (primary endpoint): 11 months for alpelisib + fulvestrant vs. 5.7 months for placebo + fulvestrant (HR 0.65; 95% CI 0.50–0.85; *p* = 0.00065) over a median follow-up of 20 months. In patients with measurable PIK3CA-mutated ABC, the ORR was 36% for patients who received alpelisib + fulvestrant vs. 16% for those who received placebo + fulvestrant (*p* = 0.0002).

The main AEs observed with alpelisib + fulvestrant vs. placebo + fulvestrant were hyperglycemia (64 vs. 10%), diarrhea (58 vs. 16%), nausea (45 vs. 22%), decreased appetite (36 vs. 10%), and rash (36 vs. 6%) (99). Discontinuation of treatment because of AEs was 5% in the alpelisib + fulvestrant group vs. 1% in the placebo + fulvestrant group. This shows that alpelisib is an option for HR-positive ABC harboring a PIK3CA mutation in the second-line setting and beyond.

## Role of Histone Deacytelase Inhibitors

DNA usually winds around histones, whose modification may lead to alterations in DNA structure, which may in turn affect transcription. In general, histone acetylation is associated with chromatin relaxation while deacetylation leads to chromatin condensation, creating a structure called heterochromatin where transcription is repressed. Because of their ability to affect DNA structure, histone deacytylase (HDAC) inhibitors are expected to have a potent role in cancer pathogenesis and progression.

HDAC1 is a prototypical deacetylase that is expressed in many tumor types, including breast cancer. Overexpression of HDAC1 in breast cancer cell line models affects ERα gene expression, leading to suppression of ERα protein.

Several trials have assessed the role of histone deacetylase inhibitors such as entinostat in the treatment of HR-positive ABC, especially as a means to overcome resistance to endocrine therapy (133).

### ENCORE 301 Trial

Encore 301 was a randomized phase II double-blind, placebo-controlled study designed to assess the impact of the addition of entinostat to exemestane on PFS. One hundred and thirty postmenopausal women with HR-positive ABC who progressed on a NSAI were randomly assigned to exemestane (25 mg daily) in addition to entinostat 5 mg or placebo weekly.

PFS was significantly longer with exemestane/entinostat than with exemestane/placebo (4.28 vs. 2.27 months, respectively; HR 0.73; *p* = 0.06). The combination was well tolerated, with the most frequent AEs consisting of fatigue, gastrointestinal disturbances, and hematologic abnormalities (100).

### NCT02115282 Trial

This trial, also identified as E2112, is an ongoing international randomized double-blind placebo-controlled phase III trial assessing the role of entinostat plus exemestane in men as well as premenopausal and postmenopausal women with HR-positive and HER2-negative ABC who have progressed after NSAI. The primary objective is PFS and OS, whereas the secondary endpoints include ORR, toxicity, and time to treatment failure.

Patients are randomly assigned in a 1:1 ratio to receive exemestane 25 mg daily plus entinostat or placebo 5 mg by mouth on days 1, 8, 15, and 22. Male participants and pre/perimenopausal women also receive goserelin 3.6 mg subcutaneously monthly.

The study is still recruiting patients. It is estimated to be completed by January 2021^4^.

### ACE Trial

This was the first phase III, double-blind, placebo-controlled study to show benefit of HDAC in HR-positive ABC. It included 362 patients with HR-positive/HER2-negative ABC who failed endocrine therapy (≤1 chemotherapy line, <4 total lines of therapy). Participants were randomly assigned to receive either chidamide (a HDAC developed in China) 30 mg twice weekly with exemestane 25 mg daily (*n* = 241), or placebo with exemestane (*n* = 121) in a 2:1 manner. The primary endpoint was PFS.

The median PFS was 7.4 months with chidamide/exemestane and 3.8 months with placebo/exemestane (HR 0.755; 95% CI 0.582–0.978; *p* = 0.0336). The most common grade ≥ 3 AEs were hematologic: neutropenia (50.8%), thrombocytopenia (27.5%), and leukopenia (18.8%) in the chidamide/exemestane group.

Further studies are needed to better study the role of HDAC inhibitors in HR-positive ABC (101).

## Role of Anti-angiogenic Agents

Angiogenesis is essential in the development, tissue invasion, and distal spread of solid tumors. Vascular endothelial growth factor (VEGF) is a major contributor to tumor angiogenesis (134). High VEGF levels in breast tumors have been associated with a decreased response to endocrine therapy. A few studies have been conducted to assess the role of anti-angiogenic agents such as bevacizumab or tyrosine kinase inhibitors such as sunitinib (135–137) and sorafenib (138, 139) in ABC.

### ECOG 2100 Trial

E2100 was an open-label, randomized, phase III trial assessing the benefit of adding bevacizumab to the treatment of HER2-negative ABC. The study demonstrated a significant improvement in PFS as the primary endpoint (HR 0.48; 95% CI 0.385–0.607; *p* < 0.0001), in addition to an improved ORR with paclitaxel plus bevacizumab compared to paclitaxel alone as initial chemotherapy (48.9 vs. 22.2%; *p* < 0.0001). Thus, the addition of bevacizumab has a substantial and robust treatment effect when added to chemotherapy (102).

Other studies such as the AVADO trial showed an improvement in response rate as well (103), but none showed an improvement in survival.

### LEA Trial

Given the proven benefit of bevacizumab when combined with chemotherapy in the treatment of breast cancer, the question arises as to its possible use with endocrine therapy in an attempt to delay the emergence of resistance. The LEA trial, a multicenter, open-label phase III trial was created to assess the combination of bevacizumab and endocrine therapy (letrozole or fulvestrant) as first-line therapy in postmenopausal patients with HR-positive/HER2-negative ABC. PFS was the primary endpoint and OS,ORR, clinical benefit rate, response duration, time to treatment failure, and safety were secondary endpoints. A total of 374 women were randomly assigned 1:1 to receive either endocrine therapy alone (letrozole 2.5 mg per day or fulvestrant 250 mg Q 4 weeks), or endocrine therapy plus bevacizumab (15 mg/kg Q 3 weeks) until disease progression or unacceptable toxicity.

Median PFS was 14.4 months with endocrine therapy alone vs. 19.3 months with endocrine therapy plus bevacizumab (HR 0.83; 95% CI 0.65–1.06; *p* = 0.126). ORR, clinical benefit rate, and response duration with endocrine therapy vs. endocrine therapy plus bevacizumab were 22 vs. 41% (*p* < 0.001), 67 vs. 77% (*p* = 0.041), and 13.3 vs. 17.6 months (*p* = 0.434), respectively. Time to treatment failure and OS were comparable in both arms. Toxicities included proteinuria and hypertension as well as elevated liver enzymes, which were higher in patients who received endocrine therapy plus bevacizumab.

The trial showed no statistically-proven benefit from the addition of bevacizumab to hormonal therapy in women with HR-positive/HER2-negative ABC in the first-line setting (104).

### TTAC-0001

TTAC-0001 is a new human monoclonal antibody developed in mouse breast cancer models targeting the VEGF receptor-2 with anti-angiogenic and antitumoral effects. In one phase I trial, the anti-tumor efficacy of the drug at different doses was assessed using ultrasonography and bioluminescence imaging, and it was compared with bevacizumab.

The higher dose of TTAC-0001 showed the strongest anti-tumor efficacy with the lowest viable tumor and micro-vessel areas and the lowest Ki-67 index. These findings suggest that it may provide a future treatment option for breast cancer (140).

## Agents Targeting the Fibroblast Growth Factor Receptor Pathway

Fibroblast growth factors are extracellular proteins that regulate cell proliferation and survival as well. They are also involved in cancer cell proliferation and angiogenesis through alteration in the RAS-MAPK and PI3K-AKT pathways. Such variations are observed in up to 25% of breast cancers.

Multiple studies have investigated the role of different fibroblast growth factor receptor (FGFR) inhibitors such as dovitinib (TKI258) (non-randomized phase II study CTKI258A2202) and lucitanib (E-3810) in the treatment of breast cancer with or without anti-estrogen therapies. Unfortunately, despite preliminary evidence of possible benefit, these agents did not meet the criteria for continuation of studies. Other studies are currently investigating additional agents: BGJ398, JNJ-42756493, and AZD4547. These studies have not yet reported results (141–143).

## Proteasome Inhibitors

The proteasome is a protease complex responsible for the cytoplasmic turnover of cellular proteins. Activity of the proteasome is essential for regulatory protein control; and its inhibition would lead to abnormal accumulation of intracellular proteins, thereby disrupting homeostasis and resulting in apoptosis of tumor cells. So, proteasome inhibitors such as bortezomib, carfilzomib and recently BU-32 (144) have been considered as potential therapeutic agents for breast cancer that is resistant to endocrine therapy, through the inhibition of mitogen-activated protein kinase phosphatase 1 (MKP-1) as well as the inhibition of signaling cascades responsible for hormone independence and anti-endocrine resistance (145).

In one phase II trial, 118 postmenopausal women with progressive disease following AI therapy were randomly assigned to receive fulvestrant alone at a dose of 500 mg or in combination with bortezomib (1.6 mg/m2 IV on days 1, 8, and 15). The primary endpoint, PFS, was 13.6% at 12 months for the fulvestrant alone group vs. 28.1% in patients treated with fulvestrant and bortezomib (*p* = 0.03). This indicates that proteasome inhibitors may be beneficial in enhancing the effect of selective ER degraders and delaying progression to endocrine therapy; however, further studies are warranted (105).

## Use of Megesterol Acetate and High-dose Estrogen After Development of Endocrine Resistance

Although there have been many novelties in the treatment of HR-positive/HER2-negative ABC progressing on hormonal therapy, historical agents such as megestrol acetate and high-dose estrogen have proven to still have a role in this domain, especially in low- and middle-income countries where newer, more expensive drugs are out of reach. For that reason, these older agents are still mentioned in guidelines for use in later lines of treatment of HR-positive ABC.

In one phase II trial, 48 postmenopausal women with HR-positive ABC, who had progressed on NSAIs with a 6 months PFS or relapsed after ≥1 year of adjuvant hormonal therapy, received megestrol acetate daily. The clinical benefit rate, which was the primary endpoint, was 40% (95% CI 25–55%]), and the median duration of clinical benefit was 10 months (95% CI 8.0–14.2). Median PFS was 3.9 (95% CI 3.0–4.8) months. Side effects were tolerable; the major side effects were deep vein thrombosis, weight gain, and fatigue with musculoskeletal pain. These results demonstrate that megestrol remains a reasonable treatment option in a cost-sensitive environment (146).

Historically speaking, there is mention of an “estrogen paradox” after estrogen deprivation, where high-dose estrogen can be used and is effective in the treatment of ABC, even after progression on prior hormonal therapy, be it tamoxifen or AIs (147).

## Pre-menopausal vs. Post-menopausal

Pre-menopausal women with ABC are a population of patients that is under-represented in clinical trials. Treatment options in this population are usually extrapolated from trials involving post-menopausal women. Pre-menopausal women are rendered menopausal via ovarian function suppression/ablation either surgically, by radiation-therapy or pharmacologically with the use of gonadotropin-hormone releasing hormone (GnRH)/luteinizing hormone releasing hormone(LHRH) agonists, after which they are treated the same way as post-menopausal patients.

Nevertheless, pre-menopausal women are different than post-menopausal women in terms of tumor biology, with a tendency for a higher grade, higher Ki67, and more advanced stage at diagnosis. Some studies have shown a higher mutational burden and a unique gene expression profile in pre-menopausal women with increased incidence of significant mutations such as ESR1, MAT2B,CTSS,DDR2, and GALANTL2 (148). In general, they also tend to have worse outcomes with an increased risk of recurrence and death, as well as poorer quality of life, with more distress, anxiety, fatigue and depression (149, 150). In most of these patients, especially those considered peri-menopausal who are closer to menopause, premature sudden menopause caused by treatment can be quite distressing with an increased risk of overall mortality and cardiovascular disease (151).

Given these differences in the young patient population, simply adding ovarian function suppression may not be the best strategy, and dedicated studies are needed for pre-menopausal women to derive an evidence-based approach for this population of patients.

Based on currently available data, ovarian function suppression in addition to endocrine therapy with or without CDK4/6 inhibitors is the standard of care. Ovarian function suppression was shown to improve patient outcomes given alone, with SERMs (152, 153), SERDs (154) and with AIs. It is mandatory for use in addition to aromatase inhibitors, given their mode of action and their stimulatory nature in case of persistence of ovarian function.

Ovarian function ablation takes place surgically with bilateral surgical salpingo-oophorectomy which is the first form of ovarian ablation tested where ovarian steroid production drops immediately and permanently, in addition to risk reduction in carriers of predisposing genes. Moreover, radiation therapy is another safe and simple technique for ovarian ablation, where females receive up to 20 Gy to the ovaries (155, 156). However, this procedure could be incomplete or delayed and requires biochemical verification of hormonal levels.

Finally, GnRH/LHRH agonists are drugs that provide safe and reversible time-limited ovarian function suppression with a side effect profile related to estrogen deprivation.

GnRH normally binds to its respective receptors in the pituitary gland leading to gonadotropin release. The mechanism by which GnRH agonists suppress ovarian function is by the prolonged activation of GnRH receptors leading to desensitization, and therefore suppressed gonadotrophin release (157). Commonly, GnRH agonists such as goserelin and leuprolide have been used for ovarian suppression in premenopausal patients with HR-positive breast cancer. OFS with GnRH/LHRH agonist was proven to be equivalent to surgical oophorectomy (158). Yet, there have been concerns regarding incomplete suppression, mostly derived from reports from patients receiving adjuvant treatment in the SOFT/TEXT early breast cancer trials. Other concerns expressed by the advanced breast cancer 4 meeting were regarding the frequency of GnRH agonist administration where despite data from a phase III non-inferiority trial showing no difference between 3-monthly vs. monthly regimens (159), some patients were not fully suppressed with the 3-monthly regimen, and had their menses back. Thereby, it was recommended that hormonal levels be taken to prove proper OFS.

Only three major studies discussed above included premenopausal women, MONALEESA-7 being the only recent phase III clinical trial targeting pre-menopausal women (90), PALOMA 3 including about 21% pre- and peri-menopausal women (87) and Monarch 2 including about 16% pre- and peri-menopausal women (91).

As a conclusion from these 3 randomized trials, ovarian function suppression with either tamoxifen, AI or fulvestrant in addition to CDK4/6 inhibitors is reasonable. In Monaleesa 7, ribociclib addition increased PFS in all subgroups with a mPFS increased from 13 months with placebo to 23.8 months in the ribociclib group with a HR of 0.55 and a *p* < 0.0001; however, mPFS in patients receiving tamoxifen/goserelin/ribociclib was 22.1 months whereas mPFS in patients receiving NSAI/goserelin/ribociclib was 27.5 months suggesting a possible benefit of AI over tamoxifen; but comparison between NSAI and tamoxifen was not intended in the study and thus such interpretation cannot be made. In PALOMA-3 and MONARCH-2, both studies included premenopausal women with HR-positive, HER2-negative ABC who progressed on ET, receiving fulvestrant + CDK4/6 inhibitor, palbociclib in PALOMA-3 and abemaciclib in MONARCH-2, both improving mPFS. To our knowledge, no randomized phase III clinical trial to date directly compares the efficacy of either fulvestrant, AI or tamoxifen, with OFS, and either can be used in first line setting. One multi-center phase II trial compared fulvestrant plus goserelin vs. anastrozole plus goserelin vs. goserelin alone in premenopausal women, with the primary endpoint being time to progression (TTP). Fulvestrant + goserelin did better with a mTTP of 16.3 vs. 14.5 months in the anastrozole + goserelin group and 13.5 months in the goserelin alone group (160). However, given the proven efficacy of fulvestrant following progression, it would probably be wise to keep it till progression.

As for other combinations used in post-menopausal women, the application of such combinations in pre-menopausal setting needs proof of benefit and multiple trials are ongoing (161). For instance, regarding the use of mTORinhibitors + non-steroidal aromatase inhibitors in addition to GnRH agonist in premenopausal patients, data is still scarce. This combination yielded higher PFS and ORR in as investigated in the BOLERO-2 trial (mentioned earlier in this article). However, concerning its application in premenopausal women (who are also subjected to ovarian suppression via GnRH agonist), the ongoing MIRACLE trial (NCT02313051)^5^ is currently investigating this option by randomizing premenopausal HR+ metastatic breast cancer patients after progression on tamoxifen to receive goserelin plus letrozole with or without everolimus. Investigators in the MIRACLE trial claim that because preclinical studies have indicated that everolimus addition to aromatase inhibitors resulted in synergistic proliferation inhibition and apoptosis induction and the BOLERO trial has yielded pleasing results, the addition of everolimus to aromatase inhibitors in premenopausal patients would be a potential viable treatment option to be evaluated.

More trials targeting treatment options for HR positive, Her2 negative ABC in pre-menopausal women are underway, most are phase I or II. Some are investigating exemestane/palbociclib in first line (FATIMA trial, NCT02592746). Others investigating combinations of CDK4/6 plus AI or fulvestrant, buparlisib plus ET, and even assessing the addition of pembrolizumab to the treatment of premenopausal women with HR-positive ABC. Results are much awaited to help answer questions and guide further treatment to what appears to be a different more aggressive tumor category.

## Discussion/Conclusion

Metastatic breast cancer is a heterogeneous disease with variable forms and different treatment options depending on tumor characteristics such as tumor biology, tumor burden and tumor molecular variations; or patient characteristics such as menopausal status, performance status, response to prior treatment if any, and timing of other treatment, access to drugs, tolerance to drugs, etc. Most studies involve post-menopausal patients, but premenopausal patients with seemingly different and at times more aggressive tumor biologies are scarcely investigated. Their therapeutic options are being extrapolated from post-menopausal studies after these women are rendered post-menopausal by OFS.

A growing number of therapeutic options make it difficult to determine the best choice of treatment in different patients. Recent years have witnessed a lot of progress in this field with a number of emerging data regarding therapeutic modalities, treatment sequence, and resistance to treatment in addition to means of overcoming such resistance.

**Figure 4:**
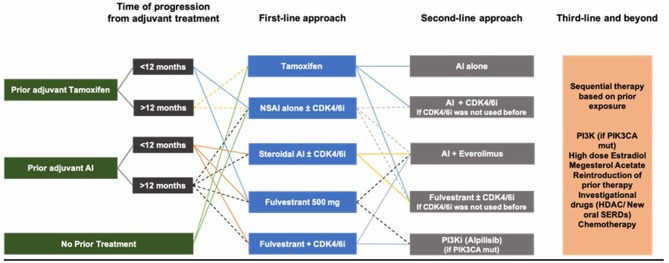
Our suggested sequence of treatment in HR+/HER2– ABC.

Based on the multiple studies and clinical trials discussed above, and although there is no international consensus on the optimal sequence of treatment, a reasonable initial approach in HR-positive ABC, after ensuring adequate ovarian function suppression in pre-/peri-menopausal women, is to start with endocrine therapy as the preferred option in the absence of visceral crisis and in the absence of proof of endocrine resistance. Initial therapy could be either tamoxifen, AI or fulvestrant. AIs remain superior to tamoxifen, whereas fulvestrant is a better option for patients previously exposed to endocrine therapy (adjuvant setting), as well as patients possibly harboring ESR1 mutations. ESR1 mutations are variable, but drugs capable of overcoming resistance caused by these mutations are underway. The use of CDK4/6 inhibitors in the first- or second-line setting for CDK4/6 inhibitor naïve patients improves PFS and quality of life and has a numerical benefit for OS for patients who have access to any CDK4/6 inhibitor, whether palbociclib, ribociclib, or abemaciclib. Treatment choice would very much be impacted by tumor burden, prior adjuvant therapy if any, and quality of life as treatment is mainly palliative. If prior adjuvant tamoxifen was used, then AI or fulvestrant are chosen in addition to LHRHa in pre-/peri-menopausal women. If prior adjuvant AI was used, then fulvestrant is a better option. If a patient has *de novo* metastatic disease with relatively high burden, then AI with CDK4/6 inhibitors are preferred (Figure 4).

In the second-line setting and beyond, it becomes difficult to find a way to overcome endocrine resistance. Fulvestrant plays some role in this regard as proven in PALOMA-3, MONALEESA-3 and MONARCH-2, and drugs targeting the mTOR/PI3K pathways have shown to be effective, as well as the new SERDs, SERM/SERD combinations.

Moreover, the addition of everolimus to AI improves survival, and everolimus/exemestane remains a valid second/third line option after prior progression on hormonal therapy (tamoxifen/NSAI/fulvestrant +/– CDK4/6 inhibitor). However, combinations with m-TOR inhibitors are not proven beneficial yet nor tested for safety in the premenopausal setting and thereby cannot be used until further data is out. More recently, PI3K inhibitors have also been shown to improve PFS, and there also seems to be a role for HDAC inhibitors. Other options need to be studied more thoroughly, such as FGFR inhibitors and proteasome inhibitors. Finally, if all hormonal therapy does not seem to be working, chemotherapy remains the last resort.

It may not be possible to establish the best sequence overall, as one size does not fit all. However, as a conclusion, in post-menopausal women, AIs plus CDK4/6 inhibitors can be suggested as first-line therapy, followed by fulvestrant or exemestane plus everolimus as second-line therapy with or without CDK4/6 inhibitors if these were not used in the first-line setting. In pre-/peri-menopausal women, definitive OFS + NSAI/tamoxifen + CDK4/6 inhibitors are valid options, followed by fulvestrant plus CDK4/6 inhibitors if CDK4/6 inhibitors were not used before. Finally, PI3K inhibitors provide another possible line of treatment for patients harboring PIK3CA mutation, before giving up on hormonal therapy.

More drugs are being developed, and more studies are underway. Clinicians await more results in this actively growing field.

## Author Contributions

RS, LJ, and HA made substantial contributions to the conception and design, acquisition of data, or analysis and interpretation of data. RS, LJ, SI, JK, MA, and HA contributed in drafting the manuscript or critically revising if for important intellectual content. All the authors approved the final manuscript submitted.

### Conflict of Interest Statement

The authors declare that the research was conducted in the absence of any commercial or financial relationships that could be construed as a potential conflict of interest.

## Footnotes

^1^https://clinicaltrials.gov/ct2/show/NCT02297438

^2^https://clinicaltrials.gov/ct2/show/NCT01864746

^3^https://clinicaltrials.gov/ct2/show/NCT02513394

^4^https://clinicaltrials.gov/ct2/show/NCT02115282

^5^Everolimus trIal for Advanced prememopausaL Breast Cancer Patients.
